# Inflammatory Profile and Osteogenic Potential of Fracture Haematoma in Humans

**DOI:** 10.3390/jcm9010047

**Published:** 2019-12-24

**Authors:** Ippokratis Pountos, Gavin Walters, Michalis Panteli, Thomas A. Einhorn, Peter V. Giannoudis

**Affiliations:** 1 Academic Department of Trauma & Orthopaedics, School of Medicine, University of Leeds, Leeds LS 2 9JT, UK; GT80@hotmail.co.uk (G.W.); michalispanteli@gmail.com (M.P.); pgiannoudi@aol.com (P.V.G.); 2 Department of Orthopaedic Surgery, NYU Langone Health, New York, NY 10016, USA; theinhorn@yahoo.com; 3 NIHR Leeds Biomedical Research Center, Chapel Allerton Hospital, LS7 4SA Leeds, West Yorkshire, Leeds LS7 4SA, UK

**Keywords:** fracture haematoma, cytokines, bone healing, mesenchymal stem cells

## Abstract

Fracture haematoma forms immediately after fracture and is considered essential for the bone healing process. Its molecular composition has been briefly investigated with our current understanding being based on animal studies. This study aims to analyse the inflammatory cytokine content of fracture haematoma in humans and determine its effect on osteoprogenitor cells. Twenty-three patients were recruited following informed consent. Peripheral blood, fracture haematoma and bone were collected. A Luminex assay on the levels of 34 cytokines was performed and autologous peripheral blood samples served as control. Mesenchymal Stem Cells (MSCs) were isolated following collagenase digestion and functional assays were performed. Gene expression analysis of 84 key osteogenic molecules was performed. Thirty-three inflammatory cytokines were found to be significantly raised in fracture haematoma when compared to peripheral serum (*p* < 0.05). Amongst the most raised molecules were IL-8, IL-11 and MMP1, -2 and -3. Fracture haematoma did not significantly affect MSC proliferation, but ALP activity and calcium deposition were significantly increased in the MSCs undergoing osteogenic differentiation. Medium supplementations with fracture haematoma resulted in a statistically significant upregulation of osteogenic genes including the EGF, FGF2 and VEGFA. This seems to be the pathway involved in the osteogenic effect of fracture haematoma on bone cells. In conclusion, fracture haematoma is found to be a medium rich in inflammatory and immunomodulatory mediators. At the same time, it contains high levels of anti-inflammatory molecules, regulates osteoclastogenesis, induces angiogenesis and the production of the extracellular matrix. It appears that fracture haematoma does not affect osteoprogenitor cells proliferation as previously thought, but induces an osteogenic phenotype.

## 1. Introduction

Bone healing is a complex process of interlinked events that leads to fracture healing [1,2,3]. It starts with the formation of haematoma, which is a rich medium characterized by abundance of a great variety of cells types, cytokines and chemokines [1]. It has been suggested that fracture haematoma is the foundation of bone healing and an essential element for the overall healing process [4]. This argument is supported clinically but also experimentally by several studies, suggesting that it contains all the required signals to initiate and actively promote the formation of new bone [4,5,6,7,8]. Implantation of fracture haematoma into soft tissues was also associated with commencement of a healing response characterized by the deposition of calcium [5]. On the contrary, some authors have shown detrimental results when fracture haematoma was removed [4,6,7]. In clinical studies, the debridement and irrigation of the fracture site within two days after fracture has shown to have the worst prognosis in terms of healing as compared to a debridement at a later stage [6]. Moreover, murine open osteotomies healed more slowly than closed fractures, an outcome attributed to the irrigation of fracture haematoma [7].

Fracture haematoma seems to be important in bone healing, but its exact cellular and molecular composition remains obscure. The limited evidence currently available mostly involves animal models. However, their fracture healing physiology is different to that of humans and therefore is not directly comparable. The aims of this study were two-fold. Firstly, it aims to analyse the inflammatory cytokine content of human fracture haematoma; secondly to determine the proliferative and osteogenic capacity of fracture haematoma compared to peripheral blood and standard culture media.

## 2. Materials and Methods

### 2.1. Patients

Between January 2015 and August 2016, patients were invited to participate in this study. Ethics committee approval was obtained (Ref number Q1206/127). Inclusion criteria included healthy adult patients admitted in our institution with closed, simple (two-part) long bone fractures requiring operative treatment. The exclusion criteria included pathological fractures, local and systemic inflammatory conditions and use of steroids or previous radiotherapy. Patients to whom surgery was delayed for more than 48 h from injury were also excluded.

In total, 23 consecutive patients (15 males; mean age of 41.3 years; range: 23 to 60 years) met the inclusion and participated in this study. The average time to surgery was 23 h (9 to 42 h). The most prevalent injury was a tibial shaft fracture (*n* = 16) followed by fibular (*n* = 3), femoral (*n* = 2) and radial fractures (*n* = 2).

### 2.2. Isolation and Preparation of Peripheral Serum and Fracture Haematoma

A total of 10 mL of peripheral venous blood was collected at the induction of surgery. Prior to any surgical intervention, 0.5 mL of fracture haematoma was also collected. Fracture haematoma collection was performed percutaneously, before any surgical intervention. Prior to the aspiration of fracture haematoma the needle position was checked on anteroposterior and lateral fluoroscopic views. The blood was collected into serum separation containers without anticoagulant and allowed to clot. It was processed within 2 h of collection and was kept on ice. The clotted blood was then centrifuged at 1500× *g* rpm for 20 min and the serum was extracted. All serum was aliquoted in Eppendorf tubes and was stored in a −80 °C freezer before use.

### 2.3. Isolation of Mesenchymal Stem Cells (MSCs)

**Figure 1:**
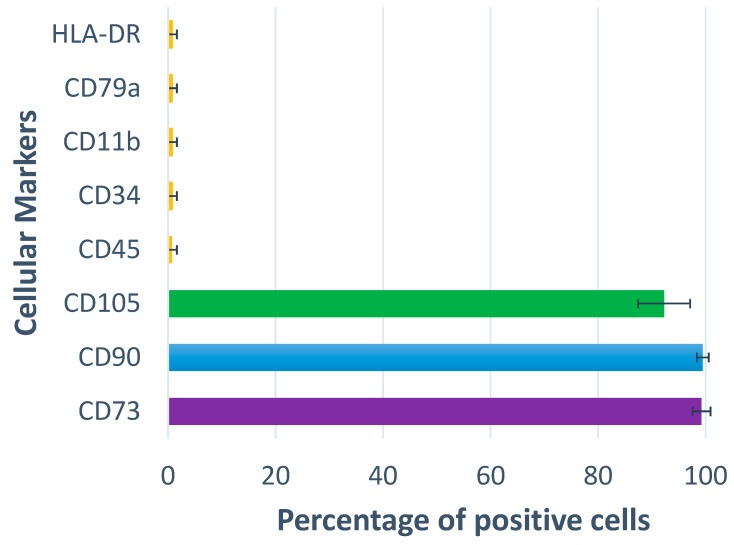
The phenotype of Mesenchymal Stem Cells used in the study. (*n* = 12).

In addition to the blood collection, bone was also harvested intra-operatively from the fracture site from 12 patients (previously described protocols) [9,10,11,12,13,14]. MSCs were isolated from bone using enzymatic digestion with collagenase 0.25% (Stem Cell Technologies, Vancouver, BC, Canada) for 4 h (37 °C, 5% CO). 20 × 10^6^ collagenase-released cells were placed into 25 cm^2^ flasks and grown to passage (p) 3. Phenotypic characterization was performed for all donors to depict the nature of cultures grown using flow cytometry (standard panel of cell surface receptors CD73, CD90 and CD105, and of haematopoetic markers CD45, CD34, CD11b, CD79α and HLA-DR) [9,10,11,12,13,14]. All cell samples were positive for the three markers, and tested negative for the negative marker cocktail containing all of the relevant haematopoetic markers (Figure 1).

### 2.4. Analysis of Proliferation

The effect of fracture haematoma on MSCs’ proliferation was assessed on cells of 12 donors according to the number of viable cells (XTT assay). Controls included cells grown on peripheral serum and standard media containing fetal calf sera. For the XTT assay 96-well-plates were used and the cells were seeded in triplicate. The cells were allowed to proliferate for 72 h and on day 3, XTT dye was added according to manufacturers’ instructions. The optical densities were proportional to the number of viable cells.

### 2.5. Analysis of Osteogenic Differentiation

Osteogenic induction was performed using p3 cells from MSCs from 10 donors using standard protocols previously described [9,10,11,12,13,14]. Briefly, the quantitative measurement of Alkaline Phosphatase (ALP) activity (Sigma, St. Louis, MO, USA, N-7653) in the cellular protein fraction was performed at day 10 following osteogenic induction. The total calcium deposition was measured at day 21 of osteogenic differentiation (Calcium Colorimetric Assay Kit, Sigma-Aldrich. MAK022, St. Louis, MO, USA). Both assays were performed under manufacturer instructions.

### 2.6. Osteogenic RT2 Profiler PCR Array

Cells from five patients were cultured under osteogenic conditions with either autologous peripheral serum or fracture haematoma. On day 10 of osteogenic differentiation, the cells were harvested and their expression of osteogenic molecules was assessed with a rtPCR array. These experiments were performed in QIAGEN laboratories and the genes examined can be found in Table 1.

### 2.7. Statistical Analysis

Assumption of normality was tested with a one-sample Kolmogorov–Smirnov test. Data are expressed as mean (standard deviation) or median (range) as appropriate. Parametric and nonparametric data were compared using the paired Student’s *t*-test and non-parametric tests respectively. The cut-off value for significance was *p* = 0.05. All calculations were done using the Statistical Package for the Social Sciences (SPSS, version 25.0, IBM, New York, NY, USA).

## 3. Results

### 3.1. Cytokines in Fracture Haematoma

Of the 37 cytokines measured in fracture haematoma and peripheral serum, IL-27 (p28 subunit), IL-32 and IL-34 were out of the detection range for the assay [Table 2]. All the rest, apart from osteocalcin, had a statistically significantly higher concentration in fracture haematoma than the respective autologous peripheral serum. The lowest statistically significant measured value was seen in IFN-y and IL-28a, both at 1.69-fold increase. The highest measured significant value was seen in IL-11, at 1698.03-fold to the peripheral serum. Other particularly high differences included the IL-8 at 235.31-fold increase, MMP2 at 13.39-fold increase, MMP1 at 13.34-fold increase and MMP3 at 12.97-fold increase.

Interestingly, the levels of anti-inflammatory molecules were raised alongside the inflammatory cytokines. IL-10, was significantly higher in fracture haematoma at a value of 5-fold (*p* < 0.001). IL-11, a potent anti-inflammatory molecule produced by cells of mesenchyme origin, was statistically significant increased compared to peripheral circulation levels (1698.03-fold difference). IL-12 and IFN that have cytoprotective and antiresorptive effect were also raised [1].

As well as performing concentration analysis, several linear regression analyses were performed to ascertain a relationship between other measured variables. Age, gender, time between fracture and sample collection, white cell counts (WCC), neutrophil counts and platelet counts were compared with cytokine concentrations in fracture haematoma and peripheral serum. Of all the variables, time between fracture and sample collection, WCC, neutrophil counts and platelet counts showed trends with cytokine concentrations. Total WCC, neutrophil counts and platelet counts were seen to correlate with several cytokines. Total WCC were seen to significantly correlate positively with BAFF (r^2^ = 0.39), INF-y (r^2^ = 0.34), IL-2 (r^2^ = 0.31), IL-19 (r^2^ = 0.29), IL-26 (r^2^ = 0.42), IL-28a (r^2^ = 0.30), IL-29 (r^2^ = 0.40), sTNF-R1 (r^2^ = 0.34), sTNF-R2 (r^2^ = 0.32), TSLP (r^2^ = 0.40) and TWEAK (r^2^ = 0.59). Similarly, significantly positive correlations were seen between neutrophil counts and fracture haematoma concentrations of BAFF (r^2^ = 0.29), IL-26 (r^2^ = 0.31) and IL-29 (r^2^ = 0.35). Platelet counts were seen to significantly correlate positively with alternative cytokines in fracture haematoma, namely sCD30 (r^2^ = 0.40), IL-20 (r^2^ = 0.44), IL-26 (r^2^ = 0.42), MMP-3 (r^2^ = 0.31) and osteopontin (r^2^ = 0.34).

### 3.2. Proliferative Potential of Fracture Haematoma

**Figure 2:**
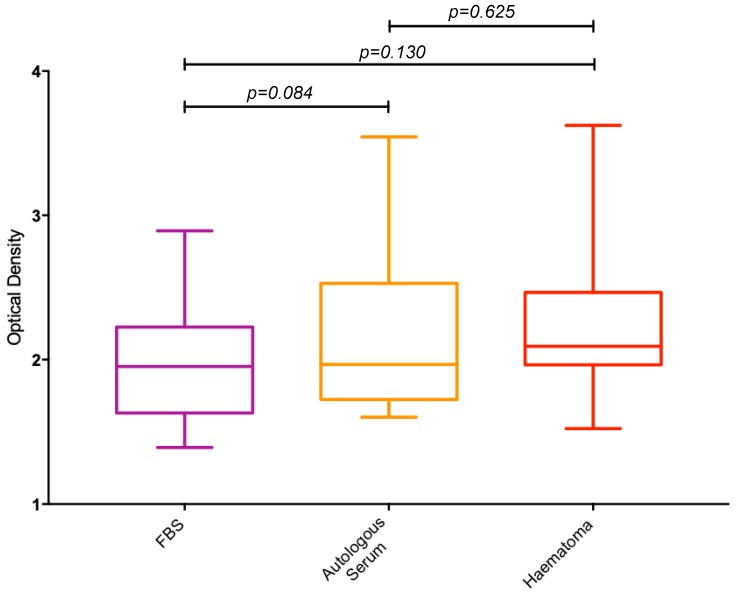
Box plots showing the ratio, expressed as a percentage, of optical densities measured from the XTT proliferation assay between FBS, autologous serum and fracture haematoma. FBS = Foetal Bovine Serum.

An XTT assay was performed on MSCs to determine the proliferative potential of fracture haematoma compared to FBS and autologous serum (Figure 2). No statistically significant differences in proliferation rates were seen between the three different culture conditions.

### 3.3. Osteogenic Potential of Fracture Haematoma

#### 3.3.1. ALP Activity Assay

**Figure 3:**
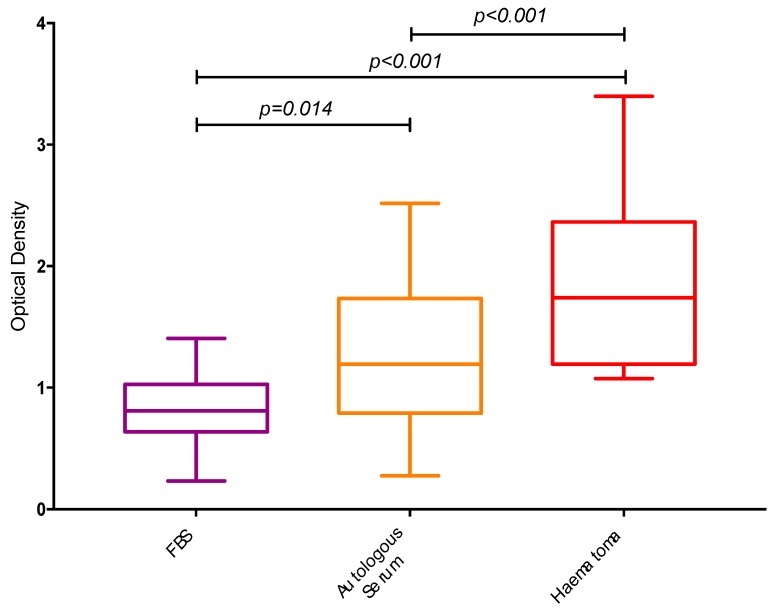
Box plots demonstrating the measured optical densities from the alkaline phosphatase activity colorimetric assay.

The measured ALP activity in osteoblasts cultured in 10% fracture haematoma was significantly higher (74%; *p* < 0.001) when compared to those cells supplemented with 10% autologous serum (Figure 3). Similarly, the ALP activity of cells complemented with fracture haematoma was 119% higher (*p* < 0.0001) compared to that of 10% FBS supplementation.

#### 3.3.2. Calcium Assay

**Figure 4:**
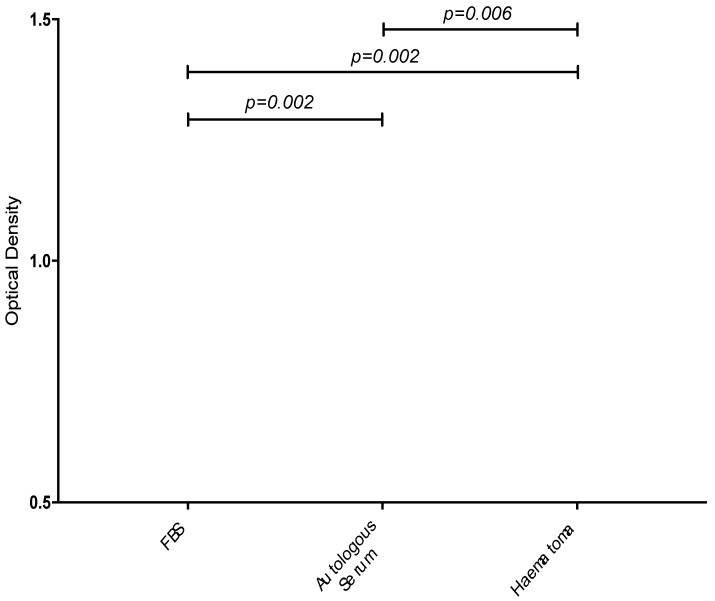
Box plots demonstrating the measured optical densities from the calcium colorimetric assay. Comparisons of calcium deposition between cells cultured in 10% FBS supplementation, 10% autologous serum and 10% fracture haematoma.

A 70% increase in calcium deposition was seen in cells cultured in fracture haematoma compared to autologous peripheral serum (Figure 4). The difference in cells cultured in human peripheral serum versus fetal animal sera was 112%.

#### 3.3.3. Gene Expression Analysis

A number of molecules demonstrated significant upregulation when exposed to fracture haematoma [Table 3]. Among the over-expressed molecules, EGF expression was upregulated by 35-fold (*p* < 0.01). VCAM1 and VEGFA were upregulated by 20.5- and 5.2-fold respectively (*p* < 0.01). Other molecules that were found upregulated included chordin (5.9-fold), SOX9 (3.8-fold), FGF2 (3.7-fold) and BMP4 (2.6-fold) (all *p* < 0.05). Statistically significant down-regulation of several molecules was also noted. BMP3 and PHEX showed the highest down-regulation with 8.0-fold and 7.2-fold respectively. BMPR1B, COL3A1 and COL14A1 were also statistically significant down-regulated.

## 4. Discussion

Our understanding on the role of fracture haematoma in humans is largely obscure and based on animal studies. This study attempts to expand our current understanding on the composition of fracture haematoma and present its direct effect on the cellular functions and expression in vitro.

Previous research has only dealt with only certain cytokines including IL-6, IL-8 IL-10, IL-12, MCP and M-CSF [15,16,17,18]. Our study is in agreement with some animal studies showing significant increases in the IL-8 and IL-10 cytokines when compared to peripheral blood [15,16,17]. Differences with previous research were also noted. Significantly higher concentrations of IL-10 and IL-12 are published in the literature; in the case of IL-12 (p70), levels of 591 ± 326 pg/mL were reported compared to a median value of 3.12 pg/mL (2.11–3.73 IQR) measured by in our study. These differences could be attributed to the method of fracture haematoma isolation, as Hauser et al. utilised urokinase to release cytokines from the fibrin matrix [16]. Hauser et al. were also unable to detect levels of IL-2 in their study. This could be potentially due to the sample preparation.

The baseline levels of these cytokines in peripheral circulation in healthy controls are largely unknown. From the limited evidence available IL-8 levels ranged between 3.00 and 3.5 pg/mL in the general population while the mean value in patients with fracture was 32 pg/mL in our study [19]. IL-11 healthy control levels were found to average at 24.6 pg/mL [20]. In our study, peripheral levels were only 1.75 pg/mL in peripheral circulation and 797 pg/mL locally in fracture haematoma. Similarly, IL-10 levels were 113.2 pg/mL in healthy controls while 9.81 pg/mL in our study [21]. As already reported by other studies, the levels of anti-inflammatory molecules like IL-10 and IL-11 reduce in some inflammatory conditions like infection and psoriasis [22,23,24]. However, further elucidation of their exact role in humans is needed.

In addition to previous published evidence, the herein study adds significant evidence on the haematoma composition and the biology of the initial fracture healing response. The presence of increased levels of several inflammatory and immunomodulatory molecules is noted. In addition to IL-6 and IL-12, other inflammatory molecules are present in haematoma including sTNF-R1, Interferons, sCD30, BAFF Chitinase-3-like 1 sTNF-R2, IL-22, IL-26, Pentaxin-3 [25]. Their presence suggests that inflammation is the landmark of the early stages of bone healing. At the same time, it also becomes evident that strong anti-inflammatory signals are present in the fracture haematoma. The levels of IL-10 (4-fold), IL-19 (2-fold), IL-27 (3-fold increase), IL-28a (2-fold increase) and IL-11 (450-fold increase); all these molecules are considered major anti-inflammatory cytokines [19,20]. Similarly to the inflammation, molecules known to promote osteoclastogenesis like TWEAK were present but at the same time IL-29 and IL-35 that are known to inhibit osteoclastogenesis were also present in increased concentrations [26,27,28,29]. Several MMPs and the TSLP known to promote collagen and extracellular matrix synthesis were also present [30,31,32]. Evidence that angiogenesis is initiated early is also obvious by the increased levels of CD163 [33,34]. Finally, IL-8, a chemo-attractant of bone progenitor cells were also present in increased concentrations [35]. The presence of the above molecules in increased concentrations highlight the complexity of the interactions of molecules of the early stages of fracture healing.

Our study also investigated the relationship between the levels of these cytokines to fracture characteristics and circulating numbers of specific cell types. Only one cytokine measured, TWEAK, exhibited a significant trend against time from fracture. One potential explanation for this decrease could be the reduction in inflammatory cell counts observed in fracture haematoma as time progresses [18,36]. In a similar manner, the significant trends observed between certain cytokines, patient peripheral WCC and neutrophil counts could be attributed to the pro-inflammatory state encountered following trauma. Platelet counts were also found to significantly correlate with sCD30, IL-20, IL-26, MMP-3 and osteopontin. Although the underlying mechanism is currently obscure, the chemotactic actions of PDGF produced by the cells could be responsible for these results.

In the second part of the study, we tried to identify what is the effect of fracture haematoma on osteoprogenitor cells in vitro. Surprisingly, fracture haematoma did not affect MSC proliferation but a positive effect of osteogenic differentiation was noted. In line with previous animal studies this effect can be attributed to the upregulated cytokine levels [37]. In particular, evidence suggests that IL-6 negatively regulates the proliferation of osteoblasts in vitro [38]. IL-22 was found to promote osteogenesis but had no effect on MSCs proliferation [39]. High levels of IL-10 were found to induce an osteogenic phenotype [40]. IFN-γ can induce the apoptosis of MSCs while IL-27 can modulate their proliferation [41]. Further elucidation of the influence of the local microenvironment on the actions of these cytokines is needed; these molecules could potentially have dual or even opposing roles in different conditions.

In an attempt to further explain the finding of the functional assays, gene expression analysis was performed. MSCs were cultured with osteogenic medium containing either autologous peripheral serum or fracture haematoma. The cell cultures with fracture haematoma showed higher expression of a number of genes implicated in osteogenesis. More specifically, a strong statistically significant expression of the EGF was noted. EGF is known to promote osteogenesis and bone healing by several studies [42]. VEGFA and FGF2 were found upregulated in the herein study. Both molecules are known to regulate and promote osteogenic differentiation [43]. SOX9, a transcription factor implicated in MSC differentiation, was also upregulated in cell exposed to fracture haematoma [44]. The significance of the down-regulated molecules is difficult to explain. BMP3 showed the highest downregulation. BMP3 suppresses osteogenic differentiation and inhibits bone formation, so its down-regulation is an indicator of enhanced osteogenesis [45]. The significance of the remaining down-regulated genes is currently unknown. It should be pointed out that BMPs, TGF beta, and Runx2 were expressed at the same level as many other cytokines. The herein study does not support any preferential role for these three classes of molecules in the healing process.

Overall, this study provides evidence on the osteogenic effect of fracture haematoma, which seems to be related to the upregulation of expression of several molecules. The study also provides evidence on the inflammatory profile of fracture haematoma in humans, describing the molecules present in fracture haematoma and their concentration. Expanding our understanding on human biology is crucial as the majority of our understanding in regards to bone healing in humans derive from animal models. Critical differences in the presences and levels of these cytokines exist between the different animal models and humans [1,15]. For example, in the study of Horst et al., IL-8 levels were 658.29 ± 84.11 pg/mL at 14 h following fracture in pigs while in the peripheral serum concentration was 148.90 ± 25.85 pg/mL. The mean value in humans was 5.243 (1.579–12.23) ng/mL, which is approximately 8-times higher. Significant differences in other fracture haematoma cytokines between animals and humans exists (reviewed and presented by Walters et al.) [1]. In addition to the levels of the cytokines, animals exhibit differences in the composition of bone, the phenotype of bone cells, several bone cell responses including their response to trauma and inflammation [46,47,48,49,50,51,52,53,54]. The mechanical forces exerted on the bone and anatomy of the bones in animals are significantly different to that of humans [46,47,48,49,50,51,52,53,54]. These differences could explain the enhanced recovery following fracture seen in small species in comparison to bigger animal models but also to humans.

Several future directions can be considered based on the results of this study. Firstly, there is need to elucidate the role and function of these inflammatory cytokines found in fracture haematoma. There is scope to further expand on the reactive oxygen species that are produced under inflammatory or ischemic conditions due to blood supply disruption vessel soft tissue damage. The mechanisms governing the oxidative stress and relation to the presence and levels of these cytokines might allow us to uncover the exact mechanism of cell damage during the fracture healing process. Correlation of the mechanical environment and the levels of these growth factors is a novel are of further research. For instance, it is known that the mechanical environment affects size and shape of callus, hence, it is likely that a different levels of growth factors are seen between stable fractures and those allowed to heal with relative stability [47,49]. Another area of interest is the inhomogeneity of fracture heamatoma, especially at the early stages, when the cells are mainly localized near the ruptured periosteum at the periphery [47,49]. Hence, exploring the role of the cells present in fracture haematoma and their contribution in the local phenotype is needed. There is vivid interest to investigate whether any of these molecules can serve as biomarker for delayed unions or non-union. Maybe the modulations of the levels of these inflammatory cytokines can provide novel strategies to enhance bone healing. In a similar manner, further studies are sought to investigate the relation of these molecules to the expression of osteogenic genes. Unfortunately, due to limited sample size this was not feasible in our study. Recent research has shown some promise, with TNF-α improving fracture healing in a murine model, but this area of biological response modification is still evolving [55]. The wide range of molecules covered in the herein study opens avenues for new molecular targets. With the advent of molecular therapy in fracture healing, this research could provide the basis for future treatments, ultimately reducing fracture healing times and the burden of fracture non-union.

## 5. Conclusions

Fracture haematoma is a rich medium in which inflammatory molecules are present, being significantly elevated compared to the levels usually met in the peripheral circulation. In addition to several inflammatory and immunomodulatory molecules, fracture haematoma seems to contain high levels of anti-inflammatory molecules, regulate osteoclastogenesis and induce angiogenesis and the production of the extracellular matrix. In-vitro functional assays highlighted that fracture haematoma does not affect the proliferation of MSCs, however, it exerts an osteogenic effect. This osteogenic induction is explained by the overexpression of several osteogenic molecules including EGF, FGFR2 and VEGFA.

## Figures and Tables

**Table 1:** List of genes examined by the osteogenic RT^2^ Profiler PCR Array.

Symbol	Description
ACVR1	Activin A receptor, type I
AHSG	Alpha-2-HS-glycoprotein
ALPL	Alkaline phosphatase, liver/bone/kidney
ANXA5	Annexin A5
BGLAP	Bone gamma-carboxyglutamate (gla) protein (Osteocalcin)
BGN	Biglycan
BMP1	Bone morphogenetic protein 1
BMP2	Bone morphogenetic protein 2
BMP3	Bone morphogenetic protein 3
BMP4	Bone morphogenetic protein 4
BMP5	Bone morphogenetic protein 5
BMP6	Bone morphogenetic protein 6
BMP7	Bone morphogenetic protein 7
BMPR1A	Bone morphogenetic protein receptor, type IA
BMPR1B	Bone morphogenetic protein receptor, type IB
BMPR2	Bone morphogenetic protein receptor, type II (serine/threonine kinase)
CALCR	Calcitonin receptor
CD36	CD36 molecule (thrombospondin receptor)
CDH11	Cadherin 11, type 2, OB-cadherin (osteoblast)
CHRD	Chordin
COL10A1	Collagen, type X, alpha 1
COL14A1	Collagen, type XIV, alpha 1
COL15A1	Collagen, type XV, alpha 1
COL1A1	Collagen, type I, alpha 1
COL1A2	Collagen, type I, alpha 2
COL2A1	Collagen, type II, alpha 1
COL3A1	Collagen, type III, alpha 1
COL5A1	Collagen, type V, alpha 1
COMP	Cartilage oligomeric matrix protein
CSF1	Colony stimulating factor 1 (macrophage)
CSF2	Colony stimulating factor 2 (granulocyte-macrophage)
CSF3	Colony stimulating factor 3 (granulocyte)
CTSK	Cathepsin K
DLX5	Distal-less homeobox 5
EGF	Epidermal growth factor
EGFR	Epidermal growth factor receptor
FGF1	Fibroblast growth factor 1 (acidic)
FGF2	Fibroblast growth factor 2 (basic)
FGFR1	Fibroblast growth factor receptor 1
FGFR2	Fibroblast growth factor receptor 2
FLT1	Fms-related tyrosine kinase 1 (vascular endothelial growth factor/vascular permeability factor receptor)
FN1	Fibronectin 1
GDF10	Growth differentiation factor 10
GLI1	GLI family zinc finger 1
ICAM1	Intercellular adhesion molecule 1
IGF1	Insulin-like growth factor 1 (somatomedin C)
IGF1R	Insulin-like growth factor 1 receptor
IGF2	Insulin-like growth factor 2 (somatomedin A)
IHH	Indian hedgehog
ITGA1	Integrin, alpha 1
ITGA2	Integrin, alpha 2 (CD49B, alpha 2 subunit of VLA-2 receptor)
ITGA3	Integrin, alpha 3 (antigen CD49C, alpha 3 subunit of VLA-3 receptor)
ITGAM	Integrin, alpha M (complement component 3 receptor 3 subunit)
ITGB1	Integrin, beta 1 (fibronectin receptor, beta polypeptide, antigen CD29 includes MDF2, MSK12)
MMP10	Matrix metallopeptidase 10 (stromelysin 2)
MMP2	Matrix metallopeptidase 2 (gelatinase A, 72kDa gelatinase, 72kDa type IV collagenase)
MMP8	Matrix metallopeptidase 8 (neutrophil collagenase)
MMP9	Matrix metallopeptidase 9 (gelatinase B, 92kDa gelatinase, 92kDa type IV collagenase)
NFKB1	Nuclear factor of kappa light polypeptide gene enhancer in B-cells 1
NOG	Noggin
PDGFA	Platelet-derived growth factor alpha polypeptide
PHEX	Phosphate regulating endopeptidase homolog, X-linked
RUNX2	Runt-related transcription factor 2
SERPINH1	Serpin peptidase inhibitor, clade H (heat shock protein 47), member 1, (collagen binding protein 1)
SMAD1	SMAD family member 1
SMAD2	SMAD family member 2
SMAD3	SMAD family member 3
SMAD4	SMAD family member 4
SMAD5	SMAD family member 5
SOX9	SRY (sex determining region Y)-box 9
SP7	Sp7 transcription factor
SPP1	Secreted phosphoprotein 1
TGFB1	Transforming growth factor, beta 1
TGFB2	Transforming growth factor, beta 2
TGFB3	Transforming growth factor, beta 3
TGFBR1	Transforming growth factor, beta receptor 1
TGFBR2	Transforming growth factor, beta receptor II (70/80kDa)
TNF	Tumor necrosis factor
TNFSF11	Tumor necrosis factor (ligand) superfamily, member 11
TWIST1	Twist homolog 1 (Drosophila)
VCAM1	Vascular cell adhesion molecule 1
VDR	Vitamin D (1,25- dihydroxyvitamin D3) receptor
VEGFA	Vascular endothelial growth factor A
VEGFB	Vascular endothelial growth factor B
ACTB	Actin, beta
B2M	Beta-2-microglobulin
GAPDH	Glyceraldehyde-3-phosphate dehydrogenase
HPRT1	Hypoxanthine phosphoribosyltransferase 1
RPLP0	Ribosomal protein, large, P0

**Table 2:** The cytokine concentrations measures in the study. Mean values in fracture haematoma and peripheral circulation.

Cytokine	Fracture Haematoma	Peripheral Serum
APRIL	357 (263–452.3) ng/mL	106.4 (67.35–136.7) ng/mL
BAFF	64.48 (40.63–72.24) ng/mL	14.25 (10.76–16.22) ng/mL
sCD30	957.3 (681.3–1442) pg/mL	683.3 (471.6–1036) pg/mL
Chitinase-3-like 1	70.36 (49.71–94.21) ng/mL	23.51 (21.07–32.24) ng/mL
GP130	281.3 (224.9–376.2) ng/mL	112.8 (99.24–155.5) ng/mL
IFNa2	81.16 (60.3–102.9) pg/mL	41.52 (35.23–47.15) pg/mL
IFNb	1017 (666.3–1586) pg/mL	179.9 (111.8–284.8) pg/mL
IFNy	42.42 (30.81–51.98) pg/mL	29.91 (24.74–34.85) pg/mL
IL-2	20.21 (14.91–24.75) pg/mL	10.16 (8.85–11.31) pg/mL
sIL-6Ra	75.51 (52.19–96.2) ng/mL	25.18 (19.85–28.59) ng/mL
IL-8	5.243 (1.579–12.23) ng/mL	0.032 (0.027–0.045) ng/mL
IL-10	42.78 (17.84–60.33) pg/mL	9.81 (7.53–11.13) pg/mL
IL-11	797 (197.8–2376) pg/mL	1.75 (1.01–5.16) pg/mL
IL-12 (p40)	104.5 (63.3–137) pg/mL	29.33 (24.09–41.73) pg/mL
IL-12 (p70)	3.12(2.07–4.19) pg/mL	1.47 (1.21–1.75) pg/mL
IL-19	32.81 (29.53–43.87) pg/mL	19.08 (17.04–23.69) pg/mL
IL-20	176.4 (116.6–217.6) pg/mL	81.39 (70.05–98.79) pg/mL
IL-22	67.41 (42.41–84.45) pg/mL	19.76 (14.48–28) pg/mL
IL-26	38.09 (21.98–47.16) pg/mL	10.43 (8.138–13.82) pg/mL
IL-27 (p28)	38.97 (24.28–47.83) pg/mL	10.43 (7.93–13.82) pg/mL
IL-28a	104.3 (86.71–123.3) pg/mL	64.35 (56.21–71.7) pg/mL
IL-29	98.41 (54.82–119.6) pg/mL	44.21 (35.62–50.5) pg/mL
IL-35	454.9 (355.9–564.6) pg/mL	219.1 (188.2–235.7) pg/mL
LIGHT	138.6 (69–267.2) pg/mL	20.87 (14.38–30.83) pg/mL
MMP-1	10.37 (6.12–16.65) ng/mL	0.94 (0.24–1.83) ng/mL
MMP-2	83.01 (36.41–107.3) ng/mL	6.10 (3.98–9.03) ng/mL
MMP-3	77.6 (38.49–122.7) ng/mL	7.54 (4.20–11.24) ng/mL
Osteocalcin	3.93 (1.67–7.10) ng/mL	2.90 (1.71–4.78) ng/mL
Osteopontin	179.4 (105.9–335.6) ng/mL	74.4 (56.48–98.75) ng/mL
Pentraxin 3	7.27 (3.59–9.50) ng/mL	0.86 (0.52–1.40) ng/mL
sTNF-R1	25.09 (15.73–31.81) ng/mL	8.58 (6.67–10.21) ng/mL
sTNF-R2	97.69 (76.1–124.3) ng/mL	16.82 (10.84–22.27) ng/mL
TSLP	105.9 (69.84–133.8) pg/mL	38.42 (33.79–46.08) pg/mL
TWEAK	1.80 (0.94–2.43) pg/mL	0.82 (0.56–0.95) pg/mL
sCD163	585.8 (471.6–1181) ng/mL	181.7 (124.3–293.6) ng/mL

**Table 3:** Gene expression of autologous cells supplemented with fracture haematoma and peripheral serum.

Gene	Fold Difference in Expression
**Upregulated**	
Epidermal Growth Factor	35.1
Vascular cell adhesion protein 1 (EGF)	20.5
GLI family zinc finger 1 (GLI)	13.0
Fms-related tyrosine kinase 1 (vascular endothelial growth factor/vascular permeability factor receptor) (FLT1)	10.8
Chordin (CHRD)	5.9
Vascular Endothelial Growth Factor-A (VEGFA)	5.2
SRY (sex determining region Y)-box 9 (SOX9)	3.8
Fibroblast growth factor 2 (FGFR2)	3.7
Bone morphogenetic protein 4 (BMP4)	2.6
**Downregulated**	
Bone morphogenetic protein 3 (BMP3)	−8.0
Phosphate regulating endopeptidase homolog, X-linked (PHEX)	−7.2
Bone morphogenetic protein receptor, type IB (BMPR1B)	−4.0
Tumor necrosis factor (ligand) superfamily, member 11 (TNFSF11)	−3.9
Collagen, type III, alpha 1 (COL3A1)	−3.2
Sp7 transcription factor (SP7)	−2.8
Collagen, type XIV, alpha 1 (COL14A1)	−2.6

## References

[1] Walters G., Pountos I., Giannoudis P.V. (2018). The cytokines and micro-environment of fracture haematoma: Current evidence. J. Tissue Eng. Regen. Med..

[2] Pountos I., Georgouli T., Pneumaticos S., Giannoudis P.V. (2013). Fracture non-union: Can biomarkers predict outcome?. Injury.

[3] Pountos I., Giannoudis P.V. (2005). Biology of mesenchymal stem cells. Injury.

[4] Grundnes O., Reikerås O. (1993). The importance of the hematoma for fracture healing in rats. Acta Orthop. Scand..

[5] Schmidt-Bleek K., Schell H., Kolar P., Pfaff M., Perka C., Buttgereit F., Duda G., Lienau J. (2009). Cellular composition of the initial fracture hematoma compared to a muscle hematoma: A study in sheep. J. Orthop. Res..

[6] Park S.H., Silva M., Bahk W.J., McKellop H., Lieberman J.R. (2002). Effect of repeated irrigation and debridement on fracture healing in an animal model. J. Orthop. Res..

[7] Park S.H., O’Connor K., Sung R., McKellop H., Sarmiento A. (1999). Comparison of healing process in open osteotomy model and closed fracture model. J. Orthop. Trauma.

[8] Grundnes O., Reikerås O. (1993). The role of hematoma and periosteal sealing for fracture healing in rats. Acta Orthop. Scand..

[9] Pountos I., Georgouli T., Henshaw K., Howard B., Giannoudis P.V. (2014). Mesenchymal Stem Cell physiology can be affected by antibiotics: An in vitro study. Cell. Mol. Biol..

[10] Pountos I., Georgouli T., Henshaw K., Bird H., Giannoudis P.V. (2013). Release of growth factors and the effect of age, sex, and severity of injury after long bone fracture. A preliminary report. Acta Orthop..

[11] Pountos I., Georgouli T., Henshaw K., Bird H., Jones E., Giannoudis P.V. (2010). The effect of bone morphogenetic protein-2, bone morphogenetic protein-7, parathyroid hormone, and platelet-derived growth factor on the proliferation and osteogenic differentiation of mesenchymal stem cells derived from osteoporotic bone. J. Orthop. Trauma.

[12] Pountos I., Giannoudis P.V., Jones E., English A., Churchman S., Field S., Ponchel F., Bird H., Emery P., McGonagle D. (2011). NSAIDS inhibit in vitro MSC chondrogenesis but not osteogenesis: Implications for mechanism of bone formation inhibition in man. J. Cell. Mol. Med..

[13] Pountos I., Georgouli T., Giannoudis P.V. (2008). The effect of autologous serum obtained after fracture on the proliferation and osteogenic differentiation of mesenchymal stem cells. Cell. Mol. Biol..

[14] Pountos I., Corscadden D., Emery P., Giannoudis P.V. (2007). Mesenchymal stem cell tissue engineering: Techniques for isolation, expansion and application. Injury.

[15] Horst K., Eschbach D., Pfeifer R., Hübenthal S., Sassen M., Steinfeldt T., Wulf H., Ruchholtz S., Pape H.C., Hildebrand F. (2015). Local inflammation in fracture hematoma: Results from a combined trauma model in pigs. Mediat. Inflamm..

[16] Hauser C.J., Zhou X., Joshi P., Cuchens M.A., Kregor P., Devidas M., Kennedy R.J., Poole G.V., Hughes J.L. (1997). The immune microenvironment of human fracture/soft-tissue hematomas and its relationship to systemic immunity. J. Trauma Acute Care Surg..

[17] Hauser C.J., Joshi P., Zhou X., Kregor P., Hardy K.J., Devidas M., Scott P., Hughes J.L. (1996). Production of interleukin-10 in human fracture soft-tissue hematomas. Shock.

[18] Hoff P., Maschmeyer P., Gaber T., Schütze T., Raue T., Schmidt-Bleek K., Dziurla R., Schellmann S., Lohanatha F.L., Röhner E. (2013). Human immune cells’ behavior and survival under bioenergetically restricted conditions in an in vitro fracture hematoma model. Cell. Mol. Immunol..

[19] Boekholdt S.M., Peters R.J., Hack C.E., Day N.E., Luben R., Bingham S.A., Wareham N.J., Reitsma P.H., Khaw K.T. (2004). IL-8 plasma concentrations and the risk of future coronary artery disease in apparently healthymen and women: The EPIC-Norfolk prospective population study. Arterioscler. Thromb. Vasc. Biol..

[20] Ren C., Chen Y., Han C., Fu D., Chen H. (2014). Plasma interleukin-11 (IL-11) levels have diagnostic and prognostic roles in patients with pancreatic cancer. Tumor Biol..

[21] Leon-Cabrera S., Arana-Lechuga Y., Esqueda-León E., Terán-Pérez G., Gonzalez-Chavez A., Escobedo G., Velázquez Moctezuma J. (2015). Reduced Systemic Levels of IL-10 Are Associated with the Severity of Obstructive Sleep Apneaand Insulin Resistance in Morbidly Obese Humans. Mediat. Inflamm..

[22] Traber K.E., Dimbo E.L., Symer E.M., Korkmaz F.T., Jones M.R., Mizgerd J.P., Quinton L.J. (2019). Roles of interleukin-11 during acute bacterial pneumonia. PLoS ONE.

[23] Cavusoglu E., Marmur J.D., Hojjati M.R., Chopra V., Butala M., Subnani R., Huda M.S., Yanamadala S., Ruwende C., Eng C. (2011). Plasma interleukin-10 levels and adverse outcomes in acute coronary syndrome. Am. J. Med..

[24] Sobhan M.R., Farshchian M., Hoseinzadeh A., Ghasemibasir H.R., Solgi G. (2016). Serum Levels of IL-10 and IL-22 Cytokines in Patients with Psoriasis. Iran. J. Immunol..

[25] Clemens M., Read A.P., Brown T. (1991). Cytokines.

[26] Hall W., Hall E., Nicola N.A. (1994). Guidebook to Cytokines and Their Receptors.

[27] Glynne Andrew J., Andrew S.M., Freemont A.J., Marsh D.R. (1994). Inflammatory cells in normal human fracture healing. Acta Orthop. Scand..

[28] Giannoudis P.V., Einhorn T.A., Marsh D. (2007). Fracture healing: The diamond concept. Injury.

[29] McCarrel T., Fortier L. (2009). Temporal growth factor release from platelet-rich plasma, trehalose lyophilized platelets, and bone marrow aspirate and their effect on tendon and ligament gene expression. J. Orthop. Res..

[30] Deuel T.F., Senior R.M., Huang J.S., Griffin G.L. (1982). Chemotaxis of monocytes and neutrophils to platelet-derived growth factor. J. Clin. Investig..

[31] Agrawal S., Ganguly S., Hajian P., Cao J.N., Agrawal A. (2015). PDGF upregulates CLEC-2 to induce T regulatory cells. Oncotarget.

[32] Chen C.F., Feng X., Liao H.Y., Jin W.J., Zhang J., Wang Y., Gong L.L., Liu J.J., Yuan X.H., Zhao B.B. (2014). Regulation of T cell proliferation by JMJD6 and PDGF-BB during chronic hepatitis B infection. Sci. Rep..

[33] Tsunoda M., Mizuno K., Matsubara T. (1993). The osteogenic potential of fracture hematoma and its mechanism on bone formation--through fracture hematoma culture and transplantation of freeze-dried hematoma. Kobe J. Med. Sci..

[34] Hasegawa T., Miwa M., Sakai Y., Nikura T., Lee S.Y., Oe K., Iwakura T., Kurosaka M., Komori T. (2012). Mandibular Hematoma Cells as a Potential Reservoir for Osteoprogenitor Cells in Fractures. J. Oral Maxillofac. Surg..

[35] Bendre M.S., Montague D.C., Peery T., Akel N.S., Gaddy D., Suva L.J. (2016). Interleukin-8 stimulation of osteoclastogenesis and bone resorption is a mechanism for the increased osteolysis of metastatic bone disease. Bone.

[36] Bordei P. (2011). Locally applied platelet-derived growth factor accelerates fracture healing. J. Bone Jt. Surg..

[37] Mizuno K., Mineo K.A.Z.U.O., Tachibana T.O.S.H.I.H.I.R.O., Sumi M.A.S.A.T.O.S.H.I., Matsubara T.S.U.K.A.S.A., Hirohata K.A.Z.U.S.H.I. (1990). The osteogenetic potential of fracture haematoma. Subperiosteal and intramuscular transplantation of the haematoma. J. Bone Jt. Surg..

[38] Kuroyanagi G., Adapala N.S., Yamaguchi R., Kamiya N., Deng Z., Aruwajoye O., Kutschke M., Chen E., Jo C., Ren Y. (2018). Interleukin-6 deletion stimulates revascularization and new bone formation following ischemic osteonecrosis in a murine model. Bone.

[39] El-Zayadi A.A., Jones E.A., Churchman S.M., Baboolal T.G., Cuthbert R.J., El-Jawhari J.J., Badawy A.M., Alase A.A., El-Sherbiny Y.M., McGonagle D. (2017). Interleukin-22 drives the proliferation, migration and osteogenic differentiation of mesenchymal stem cells: A novel cytokine that could contribute to new bone formation in spondyloarthropathies. Rheumatology.

[40] Chen E., Liu G., Zhou X., Zhang W., Wang C., Hu D., Xue D., Pan Z. (2018). Concentration-dependent, dual roles of IL-10 in the osteogenesis of human BMSCs via P38/MAPK and NF-κB signaling pathways. FASEB J..

[41] BaoB Y.Z., LvC X.L., WangB Y.Z., WangA G.F. (2017). IFN-γ induces senescence-like characteristics in mouse bone marrow mesenchymal stem cells. Adv. Clin. Exp. Med..

[42] Berendsen A.D., Olsen B.R. (2014). How vascular endothelial growth factor-A (VEGF) regulates differentiation of mesenchymal stem cells. J. Histochem. Cytochem..

[43] Hurley M.M., Adams D.J., Wang L., Jiang X., Burt P.M., Du E., Xiao L. (2016). Accelerated fracture healing in transgenic mice overexpressing an anabolic isoform of fibroblast growth factor 2. J. Cell. Biochem..

[44] Bell D.M., Leung K.K., Wheatley S.C., Ng L.J., Zhou S., Ling K.W., Sham M.H., Koopman P., Tam P.P., Cheah K.S. (1997). SOX9 directly regulates the type-II collagen gene. Nat. Genet..

[45] Kokabu S., Gamer L., Cox K., Lowery J., Tsuji K., Raz R., Economides A., Katagiri T., Rosen V. (2012). BMP3 suppresses osteoblast differentiation of bone marrow stromal cells via interaction with Acvr2b. Mol. Endocrinol..

[46] Checa S., Prendergast P.J., Duda G.N. (2011). Inter-species investigation of the mechano-regulation of bone healing: Comparison of secondarybone healing in sheep and rat. J. Biomech..

[47] Haffner-Luntzer M., Hankenson K.D., Ignatius A., Pfeifer R., Khader B.A., Hildebrand F., van Griensven M., Pape H.C., Lehmicke M. (2019). A Review of Animal Models of Comorbidities in Fracture-Healing Research. J. Orthop. Resh..

[48] Torricelli P., Fini M., Giavaresi G., Borsari V., Carpi A., Nicolini A., Giardino R. (2003). Comparative interspecies investigation on osteoblast cultures: Data on cell viability and syntheticactivity. Biomed. Pharmacother..

[49] Schell H., Duda G.N., Peters A., Tsitsilonis S., Johnson K.A., Schmidt-Bleek K. (2017). The haematoma and its role in bone healing. J. Exp. Orthop..

[50] Yang X., Yip J., Anastassiades T., Harrison M., Brockhausen I. (2007). The action of TNFalpha and TGFbeta include specific alterations of the glycosylation of bovine and human chondrocytes. Biochim. Biophys. Acta.

[51] Nemoto K., Pilbeam C.C., Bilak S.R., Raisz L.G. (1997). Molecular cloning and expression of a rat prostaglandin E2 receptor of the EP2 subtype. Prostaglandins.

[52] Kasugai S., Oida S., Iimura T., Arai N., Takeda K., Ohya K., Sasaki S. (1995). Expression of prostaglandin E receptor subtypes in bone: Expression of EP2 in bone development. Bone.

[53] Jiang Y., Lin H., Tuan R.S. (2017). Overview: State of the Art and Future Prospectives  for Cartilage Repair. Cartilage.

[54] Malda J., de Grauw J.C., Benders K.E., Kik M.J., van de Lest C.H., Creemers L.B., Dhert W.J., van Weeren P.R. (2013). Of mice, men and elephants: The relation between articular cartilage thickness and body mass. PLoS ONE.

[55] Wahl E.C., Aronson J., Liu L., Fowlkes J.L., Thrailkill K.M., Bunn R.C., Skinner R.A., Miller M.J., Cockrell G.E., Clark L.M. (2010). Restoration of regenerative osteoblastogenesis in aged mice: Modulation of TNF. J. Bone Miner. Res..

